# Ferroptosis regulatory networks and precision interventions in autoimmune hepatitis: comparison with cholestatic diseases

**DOI:** 10.3389/fimmu.2026.1874855

**Published:** 2026-08-11

**Authors:** Wenhui Zeng, Jiabing Wang, Yan Xu, Shuzhou Wu, Cixiang Chen, Shanrong Zhang, Bingli Hu, Junsong Ye

**Affiliations:** 1 Subcenter for Stem Cell Clinical Translation, First Affiliated Hospital of Gannan Medical University, Ganzhou, Jiangxi, China; 2 School of Rehabilitation Medicine, Gannan Medical University, Ganzhou, Jiangxi, China; 3 Ganzhou Key Laboratory of Stem Cell and Regenerative Medicine, Ganzhou, Jiangxi, China; 4 School of Pharmacy, Gannan Medical University, Ganzhou, Jiangxi, China; 5 First Clinical Medical College, Gannan Medical University, Ganzhou, Jiangxi, China; 6 Key Laboratory of Prevention and treatment of cardiovascular and cerebrovascular diseases, Ministry of Education, Gannan Medical University, Ganzhou, Jiangxi, China; 7 Jiangxi Provincial Key Laboratory of Tissue Engineering, Gannan Medical University, Ganzhou, Jiangxi, China; 8 Clinical Medicine Research Center, First Affiliated Hospital of Gannan Medical University, Ganzhou, Jiangxi, China

**Keywords:** autoimmune diseases, ferroptosis, liver diseases, pathogenesis, therapeutic strategies

## Abstract

Ferroptosis is a regulated form of cell death driven by iron-dependent lipid peroxidation, characterized by disruption of iron homeostasis, imbalance in the antioxidant system, and accumulation of lipid peroxides. Its core execution machinery is highly conserved across tissues, whereas disease-specific upstream nodes dictate pathway activation and progression in different pathological contexts. In autoimmune hepatitis (AIH), ferroptosis is governed by a “bidirectional imbalance” between enhanced pro-death signals and compromised anti-death protective mechanisms, further modulated by endocrine and metabolic factors. This imbalance collectively lowers the ferroptosis threshold in hepatocytes, amplifying their susceptibility to ferroptotic cell death. Although hepatocyte ferroptosis has been observed in experimental cholestasis models, these models do not directly represent the pathological process of primary biliary cholangitis (PBC). In PBC patients, recent studies have identified ferroptosis signals in monocyte-derived macrophages, but ferroptosis in biliary epithelial cells remains unconfirmed. In primary sclerosing cholangitis (PSC), despite elevated oxidative stress markers, the absence of core ferroptosis executioner molecules precludes any definitive link to ferroptosis. This review comprehensively summarizes the ferroptosis regulatory network and its disease-specific manifestations across autoimmune liver diseases, with a focus on AIH-specific nodes as potential precision intervention targets. We also propose a phase-based therapeutic framework and discuss safety considerations, as well as key challenges for clinical translation.

## 1 Introduction

Autoimmune hepatitis (AIH) is a typical T cell-mediated immune disease, whose pathological features are mainly characterized by CD4^+^/CD8^+^ T cell infiltration and the release of pro-inflammatory factors, leading to hepatocyte injury (1, 2). In recent years, ferroptosis, a form of cell death driven by iron-dependent lipid peroxidation, has been confirmed to play a pivotal role in the pathogenesis of AIH (3). Clinical data have revealed elevated serum ferritin levels that correlate with fibrosis stage, along with hepatic iron deposition and lipid peroxidation products in liver tissues, suggesting a ferroptosis-permissive microenvironment (4, 5). Mechanistic studies have demonstrated the presence of specific dysregulation of the ferroptosis regulatory network in AIH: firstly, liver-specific highly expressed miR-122-5p weakens cysteine uptake and glutathione (GSH) synthesis by targeting and inhibiting solute carrier family 7 member 11 (SLC7A11), resulting in decreased antioxidant defense capacity; secondly, A20 (TNFAIP3) dysfunction impairs the A20-mediated ubiquitination and degradation of Kelch-like ECH-associated protein 1 (KEAP1), thereby suppressing nuclear factor erythroid 2-related factor 2 (Nrf2) activation and compromising antioxidant defense; in addition, the dysfunction of the fibroblast growth factor 4 (FGF4) - CDGSH iron sulfur domain 3 (CISD3) protective axis further aggravates the production of mitochondria-derived reactive oxygen species (6–8). These mechanisms collectively lower the ferroptosis threshold of hepatocytes, constituting the molecular signature of AIH that distinguishes it from other liver diseases.

In contrast, primary biliary cholangitis (PBC) and primary sclerosing cholangitis (PSC), as cholestatic autoimmune liver diseases, exhibit different patterns of ferroptosis involvement compared with AIH. In PBC, the accumulation of hydrophobic bile acids can trigger oxidative stress and lipid peroxidation. Clinical patients have reported elevated serum levels of malondialdehyde (MDA) and 8-isoprostane (8-iso-PGF2α) that correlate with disease staging, and 4-hydroxynonenal (4-HNE) adduct deposition can be observed in liver tissues; recent single-cell transcriptomics combined with multiplex immunofluorescence has revealed that monocyte-derived macrophages (MoMFs) in PBC livers exhibit ferroptosis signaling centered on the calpain/acyl-CoA synthetase long-chain family member 4 (ACSL4) axis, and its targeting alleviates liver injury in PBC-like mouse models (9). However, although these oxidative stress markers are consistent with the pathological characteristics of ferroptosis, there is still a lack of direct verification for ferroptosis-specific markers in PBC patients (10, 11). For PSC, although ferroptosis may theoretically exacerbate inflammatory responses through the release of damage-associated molecular patterns (DAMPs) (12), to date, no direct evidence has confirmed the occurrence of ferroptosis in biliary epithelial cells of PSC patients, and existing research efforts are still mainly focused on cellular senescence and fibrotic progression (13).

This review comprehensively elaborates the core molecular mechanisms of ferroptosis, focuses on dissecting the bidirectional regulatory network of ferroptosis and its specific functional nodes in AIH, and compares the differential evidence of pathological features between PBC and PSC in detail. On this basis, we further discuss a phase-adapted therapeutic framework targeting distinct ferroptosis-related nodes at different disease stages, along with safety considerations and key challenges for clinical translation, aiming to provide a theoretical basis for the precise prevention and treatment of autoimmune liver diseases.

## 2 Mechanisms of ferroptosis

**Figure 1:**
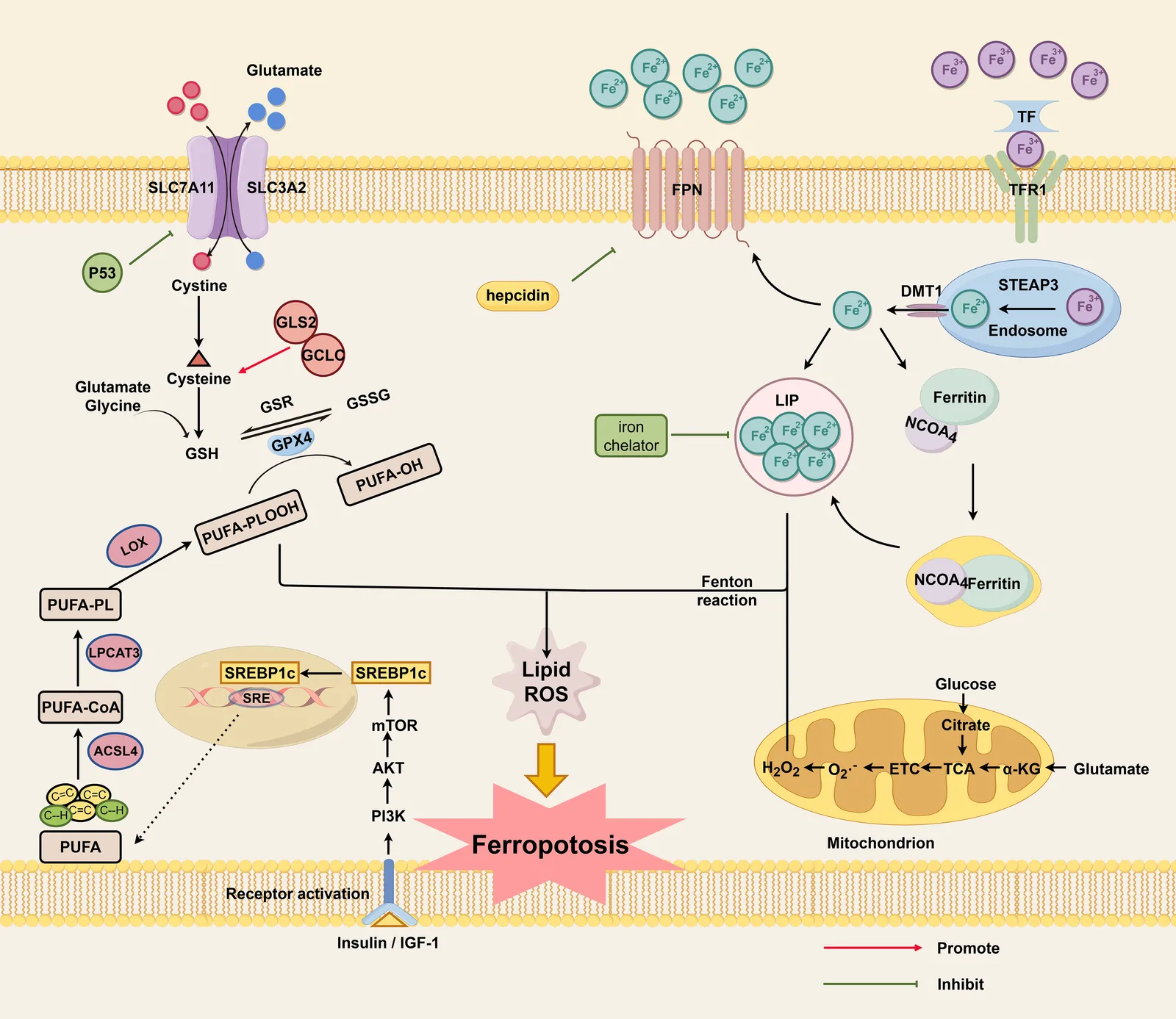
Ferroptosis regulatory network. This figure outlines three core processes and upstream regulation. (1) Antioxidant defense: System Xc⁻ imports cystine for GSH synthesis; GPX4 uses GSH to eliminate lipid peroxides. (2) Iron-driven damage: TFR1 imports Fe²^+^, Fenton reaction generates ·OH, initiating peroxidation; ferritin stores iron, NCOA4 mediates ferritinophagy to release iron. (3) Membrane peroxidation: ACSL4/LPCAT3 incorporate PUFAs into phospholipids, which are peroxidized by LOXs or ·OH. Upstream: insulin/IGF-1 via SREBP1c promotes PUFA synthesis; glucose via TCA-ETC yields H_2_O_2_. This figure was created with Figdraw.

Unlike the four classical forms of cell death, ferroptosis is characterized by dysregulated iron metabolism, imbalance in the antioxidant system, and accumulation of lipid peroxides (**Figure 1**) (14). Among these, disruption of iron homeostasis elevates intracellular labile iron (Fe²^+^) levels; Fe²^+^ subsequently catalyzes the Fenton reaction to generate reactive oxygen species (ROS), including hydroxyl radicals (•OH) and hydrogen peroxide (H_2_O_2_), thereby promoting lipid peroxidation, leading to the lethal accumulation of lipid peroxides and ultimately triggering ferroptosis (15). Within the amino acid metabolic framework of ferroptosis, GSH functions as a critical antioxidant strictly dependent on cysteine availability. Intracellular cysteine is acquired via the cystine/glutamate antiporter System Xc⁻-mediated uptake of extracellular cystine or endogenous generation through the transsulfuration pathway (16). Notably, hepatocytes predominantly rely on System Xc⁻ for extracellular cysteine acquisition; its inhibition compromises GSH biosynthesis and attenuates glutathione peroxidase 4(GPX4) activity (17). As an essential GPX4 cofactor, GSH enables the enzymatic reduction of toxic lipid peroxides to non-toxic lipid alcohols, thereby terminating peroxidation chain reactions and suppressing ferroptosis. Consequently, ferroptosis is triggered by either inadequate GPX4-mediated detoxification or excessive lipid peroxidation, ultimately culminating in cell death (18).

### 2.1 Iron metabolism disruption

Iron is an essential trace element in living organisms, extensively involved in various critical physiological processes including energy metabolism, oxygen transport, mitochondrial respiratory chain electron transfer, immune regulation, heme synthesis, and erythropoiesis (19–21). In the body, iron primarily exists in two ionic states, Fe²^+^ and Fe³^+^, and is transported and stored between cells and tissues through complex and finely regulated metabolic pathways (22). Extracellular Fe³^+^ binds to transferrin (TF), then the complex binds to transferrin receptor protein 1 (TFR1) and enters cells via endocytosis; within endosomes, Fe³^+^ is reduced to Fe²^+^ and subsequently transported to the cytosol by divalent metal transporter 1 (DMT1) (23). A portion of cytoplasmic Fe²^+^ enters the labile iron pool (LIP), serving as a readily available source for metabolic utilization, while excess Fe²^+^ is transported into ferritin and oxidized to Fe³^+^ for safe storage. The remaining Fe²^+^ is exported out of cells through ferroportin (FPN) and subsequently oxidized to Fe³^+^ by membrane-bound multicopper oxidases, enabling its binding to transferrin for systemic circulation (24, 25). Importantly, FPN-mediated iron export is subject to tight endocrine regulation by the hepatic hormone hepcidin. Hepcidin binds to FPN, triggering its internalization and lysosomal degradation, consequently blocking iron export and promoting intracellular iron retention within the LIP. Therefore, the hepcidin-FPN axis serves as a systemic checkpoint that counterbalances TFR1-mediated iron import; its dysregulation, predominantly driven by hepcidin upregulation, tilts the balance toward net iron accumulation, exacerbates LIP loading, and sensitizes cells to ferroptosis (26).

Building upon this regulatory framework, disordered iron metabolism is a core driver of ferroptosis, with key mechanisms including iron overload and the Fenton reaction. Iron overload leads to abnormally elevated intracellular Fe²^+^ levels. When the iron content in the LIP exceeds homeostatic thresholds, excess Fe²^+^ reacts with H_2_O_2_ via the Fenton reaction, generating •OH. These radicals initiate chain reactions of lipid peroxidation, causing structural damage to cell membranes and ultimately leading to cell death (27). Studies show that iron chelators can inhibit ferroptosis by reducing intracellular free iron concentrations. Additionally, ferritin can release iron through nuclear receptor coactivator 4 (NCOA4)-mediated autophagy, thereby promoting ferroptosis (28, 29). Thus, ferritinophagy can be considered an upstream regulatory mechanism of ferroptosis, influencing cell fate by modulating intracellular iron levels and lipid peroxidation.

The liver is the central organ regulating systemic iron metabolism, synthesizing hepcidin and transferrin (30). In AIH patients, serum hepcidin is significantly reduced and correlates negatively with alkaline phosphatase (ALP) (31). This paradox, defined as the discrepancy between the expected IL-6/STAT3-mediated upregulation of hepcidin and the observed decrease in serum hepcidin in AIH patients, may be explained by the dominance of anemia/hypoxia-driven signals that redirect iron metabolic priorities, thereby counteracting or diminishing the IL-6/STAT3-mediated hepcidin upregulation (32). The role of glucocorticoid therapy remains unclear; AIH patients on immunosuppression maintain lower hepcidin than other chronic liver disease patients over two years (31), but direct evidence that corticosteroids alter hepcidin expression in AIH livers is lacking. These mechanisms require further investigation.

### 2.2 Imbalance in the amino acid antioxidant system

Amino acid metabolism, especially cystine transport and GSH synthesis, plays a central role in ferroptosis regulation. The System Xc⁻, a key component, is a Na^+^ -independent transmembrane protein formed by the light chain SLC7A11 and heavy chain SLC3A2 (33). Its main function is to transport one extracellular cystine molecule into the cell and one intracellular glutamate molecule out of the cell in a 1:1 ratio. The intracellular cystine taken up is a key precursor for GSH synthesis (34). GSH, a vital cellular antioxidant composed of glutamate, cysteine, and glycine, is involved in the antioxidant defense system through GPX4 (35). It helps eliminate toxic lipid peroxides and is oxidized to oxidized glutathione (GSSG) in the process (34, 36).

The occurrence of ferroptosis is closely associated with GSH depletion and the consequent loss of GPX4 activity. GSH depletion impairs GPX4-mediated detoxification of lipid peroxides, leading to their accumulation and ultimately triggering cell death. Functional inhibition of System Xc⁻ restricts cystine uptake, thereby hindering GSH synthesis, compromising cellular antioxidant capacity, and inducing ferroptosis. Additionally, glutaminase 2 (GLS2) and the catalytic subunit of glutamate-cysteine ligase (GCLC) represent critical regulators of GSH metabolism; GLS2 facilitates glutamate production, while GCLC catalyzes the rate-limiting step in GSH biosynthesis (37). Upregulation of GLS2 and GCLC enhances GSH synthetic capacity and bolsters cellular antioxidant defenses, thereby exerting protective effects against ferroptosis (38, 39). Collectively, these mechanisms suggest that targeting the expression or activity of these key molecules holds promise for developing novel therapeutic strategies for ferroptosis-associated diseases.

### 2.3 Accumulation of lipid peroxides

The accumulation of lipid peroxides is a hallmark feature of ferroptosis (40). This process primarily occurs within phospholipid molecules rich in polyunsaturated fatty acids (PUFAs) located in cellular and organellar membranes. The structure of PUFAs contains multiple oxidizable carbon-carbon double bonds and highly reactive carbon-hydrogen bonds, rendering them highly susceptible to peroxidation. During lipid metabolism, PUFAs are converted into PUFA-CoA under the catalysis of ACSL4. Subsequently, lysophosphatidylcholine acyltransferase 3 (LPCAT3) esterifies and incorporates them into membrane phospholipids, forming PUFA-containing phospholipids (PUFA-PLs), which serve as critical substrates for lipid peroxidation during ferroptosis (41). Furthermore, the composition of PUFA-PLs can be modulated by endogenous hormonal influences. For instance, estrogens have been shown to exert membrane-stabilizing and antioxidant effects that may counteract lipid peroxidation (42).

Beyond such compositional regulation, the availabilityb of PUFA substrates is also tightly controlled at the level of *de novo* synthesis by upstream metabolic hormones. Specifically, insulin/IGF-1 sign aling activates the PI3K/AKT/mTORC1 pathway, which in turn promotes the nuclear translocation of the transcription factor SREBP1c and upregulates the expression of fatty acid synthase (FASN) and stearoyl-CoA desaturase 1 (SCD1), thereby driving *de novo* lipogenesis that supplies abundant PUFA precursors for membrane phospholipids (42, 43). This regulatory axis directly couples systemic metabolic states, such as hyperinsulinemia, to ferroptosis sensitivity.

Peroxidation of PUFA-PLs proceeds via two major pathways: the enzyme-catalyzed pathway and the non-enzymatic pathway. In the enzyme-catalyzed pathway, lipoxygenases (LOXs) directly catalyze the oxidation of PUFA-PLs, generating lipid hydroperoxides (PUFA-PLOOH) (16). In the non-enzymatic pathway, excess Fe²^+^ generates ROS, such as •OH, via the Fenton reaction. •OH abstracts a hydrogen atom from PUFA-PLs, forming lipid radicals (PUFA-PL•). Oxygen (O_2_) then oxidizes PUFA-PL• to lipid peroxyl radicals (PUFA-PLOO•). These radicals can further abstract a hydrogen atom from adjacent PUFA-PL molecules, initiating a free radical chain reaction that ultimately yields lipid peroxides, such as PUFA-PLOOH (44, 45). The lipid peroxidation end products generated by this chain reaction, such as MDA and 4-HNE, exhibit strong cytotoxicity. These compounds can disrupt the integrity and function of biological membranes, induce organellar dysfunction, and ultimately drive ferroptosis (46).

## 3 Regulatory network of ferroptosis signaling pathways and its clinical significance

Ferroptosis is precisely modulated by a dual signaling network: antiferroptotic pathways eliminate lipid peroxides or enhance cellular antioxidant capacity, whereas pro-ferroptotic pathways promote lipid peroxidation or iron accumulation. Dysregulation of these pathways contributes to the pathogenesis of multiple liver diseases, such as hepatocellular carcinoma (HCC), autoimmune hepatitis (AIH), nonalcoholic steatohepatitis (NASH), nonalcoholic fatty liver disease (NAFLD), and acute liver failure (ALF) (**Table 1**). In-depth dissection of these regulatory mechanisms not only helps elucidate the molecular pathological basis of diverse liver disorders but also provides a vital theoretical foundation for developing precise therapeutic strategies targeting ferroptosis.

**Table 1:** Ferroptosis-related signaling pathways and their roles in liver diseases.

Signal pathway	Mechanism	Effect on Ferroptosis	Role in liver diseases	Study type	Experimental model	Evidence level	Direct relevance to AIH	Ref
GPX4-dependent pathway	GPX4, a key reductase, utilizes GSH to reduce lipid peroxides to alcohols, directly detoxifying lethal lipid peroxides	Inhibit	AIH: GPX4 downregulation induces ferroptosis; Ferrostatin-1 (Fer-1) reverses injury in AIH models	Experimental + clinical	Con A-induced AIH mouse model; AIH patient samples	Direct (animal model + clinical observation)	High	(16, 47)
FSP1-CoQ10-NADPH	FSP1 regenerates the lipophilic radical trapping antioxidant CoQ10H2 from CoQ10, independently of GPX4.	Inhibit	HCC: Upregulated FSP1 confers resistance to sorafenib-induced ferroptosis and promotes tumor growth.	Experimental	HCC cell lines	Indirect (other diseases)	Low	(48, 49)
Nrf2/HO-1	Nrf2 translocates to activate the HO-1 gene, thereby enhancing cellular antioxidant capacity.	Inhibit	AIH: Activated Nrf2 suppresses ferroptosis and alleviates liver injury.	Experimental	S100-induced AIH mouse model	Direct (animal model)	High	(7, 50)
GCH1/BH4	GCH1 synthesizes BH4, which acts as a free radical scavenger to protect lipid membranes against peroxidation.	Inhibit	NASH:The axis inhibits ferroptosis by selectively protecting phospholipids with two PUFA tails from lipid peroxidation.	Experimental	NASH mouse models; hepatocyte cultures	Indirect (other diseases)	Low	(51)
ACSL4-dependent pathway	ACSL4 catalyzes the acylation of PUFAs to generate oxidation-prone phospholipids, thereby promoting lipid peroxidation.	Promote	HCC:Upregulated ACSL4 acts as an executor of ferroptosis, and agents such as curcumin induce death of hepatocellular carcinoma cells by upregulating ACSL4.	Experimental	HCC cell lines	Indirect (other diseases)	Moderate	(52, 53)
p53-SAT1	p53 transcriptionally activates SAT1, enhancing polyamine catabolism and ROS production, consequently promoting lipid peroxidation and ferroptosis execution.	Promote	HCC: The WWOX protein can significantly induce ferroptosis in HCC cells by regulating the p53/SAT1 axis	Experimental	HCC cell lines	Indirect (other diseases)	Low	(54)
HIF-2α	HIF-2α induces the expression of TFR1 and iron storage proteins, increases intracellular Fe²^+^ levels, and promotes the Fenton reaction.	Promote	NAFLD:In hepatocyte-specific HIF-2α-deficient mice, hepatic iron accumulation, lipid peroxidation, and NAFLD severity were significantly attenuated.	Experimental	Hepatocyte-specific HIF-2α-deficient mouse models	Indirect (other diseases)	Low	(55)
NCOA4	NCOA4 selectively degrades ferritin and releases free iron to promote the Fenton reaction.	Promote	ALF:HNF4A negatively regulates NCOA4, thereby suppressing ferritinophagy.	Experimental	ALF mouse models	Indirect (other diseases)	Low	(56)

Nrf2 note: Protective in acute AIH, but sustained activation in HCC may counteract ferroptosis and promote tumor survival (dual role).

## 4 Ferroptosis in autoimmune liver diseases: evidence and molecular mechanisms

### 4.1 Ferroptosis and autoimmune hepatitis: evidence of hepatocyte injury involvement

This section summarizes the current evidence linking ferroptosis to AIH, encompassing proof-of-concept mechanistic findings from animal models and hypothesis-generating signals from clinical observations, alongside mechanistic insights derived from both AIH-specific models and other disease contexts.

#### 4.1.1 AIH disease characteristics and animal models

AIH is an immune-mediated chronic progressive liver disease serologically characterized by elevated serum transaminases and immunoglobulin G (IgG) levels, along with positivity for antinuclear antibodies (ANA) or anti-smooth muscle antibodies (ASMA). Histologically, it presents as interface lymphoplasmacytic infiltration (57). This condition affects individuals across all age groups from childhood to old age, where persistent liver injury ultimately progresses to cirrhosis and liver failure. Although the pathogenesis remains incompletely understood, two animal models have provided key mechanistic clues (58, 59): The concanavalin A (Con A) model recapitulates T-cell-mediated pathology via activation of CD4^+^/CD8^+^ T cells, natural killer (NK) cells, and phagocytes (60); The S100 protein-induced model has linked the Nrf2/HO-1 pathway to oxidative stress and iron metabolism dysregulation (47). However, it is important to note that most evidence for ferroptosis in AIH currently derives from these animal models and indirect biomarkers; direct evidence in human hepatocytes remains limited.

#### 4.1.2 Molecular coupling mechanisms between immune response and ferroptosis

Building upon these animal model observations, recent studies have begun to elucidate the molecular pathways linking immune activation to hepatocyte ferroptosis in AIH. A growing body of circumstantial evidence points to a molecular coupling between autoimmune response and ferroptosis in AIH, although direct causal proof in human disease remains to be established. Evidence from tumor immunology demonstrates that activated CD8^+^ T cells release IFN-γ, which downregulates SLC7A11—a critical component of System Xc⁻—on target cells via the JAK-STAT signaling pathway. SLC7A11 suppression blocks cystine uptake, resulting in GSH depletion and GPX4 inactivation, ultimately triggering ferroptosis (61). In Con A-induced AIH mouse models, elevated serum IFN-γ levels coincide with hepatic downregulation of SLC7A11 and GPX4, accompanied by increased MDA and free iron accumulation (47). Beyond this cytokine, the expression of multiple inflammatory cytokines is altered, and some of these factors have been reported to be associated with ferroptosis regulation in other disease contexts (**Table 2**) (2), suggesting a broader immune–ferroptosis interface within the hepatic inflammatory microenvironment. However, mechanistic studies reveal that indoleamine 2,3-dioxygenase 1 (IDO1), rather than IFN-γ per se, serves as the key regulator of SLC7A11 expression—IDO1 promotes hepatocyte ferroptosis by inducing nitrosative stress (RNS) to suppress SLC7A11 (62). Although IFN-γ upregulates IDO1, its direct role in downregulating GPX4 or SLC7A11 lacks specific experimental validation in AIH (63, 64). This mechanistic framework suggests that adaptive immune responses may link to hepatocyte ferroptosis through an IDO1-mediated indirect pathway, yet the direct involvement of the IFN-γ→SLC7A11 axis in AIH requires further verification via hepatocyte-specific IFN-γR knockout or pathway inhibition studies.

**Table 2:** Inflammatory cytokine alterations in the Con A-induced AIH model and their potential association with ferroptosis.

Inflammatory cytokines	Primary immune source	Change in Con A-induced AIH model	Known ferroptosis-related function (from other disease models)	Ref
IL-6	CD4^+^ T cells, Kupffer cells	Increase	Pro-ferroptotic: In intermittent hypoxia-associated NAFLD, macrophage-derived IL-6 upregulates MARCH3, which ubiquitinates and degrades GPX4, thereby promoting hepatocyte ferroptosis and lipid accumulation.	(65, 66)
IL-1β	NKT cells, Kupffer cells	Increase	Pro-ferroptotic: In chondrocytes, IL-1β upregulates Sp1, which transcriptionally activates ACSL4, thereby directly inducing ferroptosis.	(67, 68)
TNF-α	Kupffer cells, CD4^+^ T cells, NKT cells	Increase	Pro-ferroptotic: In LPS-induced liver injury models, LPS-activated macrophages secrete TNF-α, which triggers hepatocyte ferroptosis.	(69, 70)
IFN-γ	CD8^+^ T cells, CD4^+^ T cells, NK cells, NKT cells	Increase	Pro-ferroptotic: In HCC, IFN-γ released by CD8^+^ T cells activates JAK/STAT signaling, which transcriptionally represses SLC7A11 and SLC3A2, thereby depleting glutathione and promoting lipid peroxidation and ferroptosis.	(71, 72)
IL-2	CD4^+^ T cell	Increase	Pro-ferroptotic: In the tumor microenvironment, PGE2 impairs IL-2 sensing by downregulating the IL-2Rγc chain in CD8^+^ TILs, thereby causing defective IL-2-mTOR adaptation and PGC1α transcriptional repression, leading to oxidative stress and ferroptotic cell death.	(73, 74)
IL-17	CD4^+^ T cells, NKT cells	Increase	Pro-ferroptotic: In chondrocytes, IL-17A activates lipid peroxidation and suppresses GPX4, leading to ferroptosis.	(75, 76)
IL-18	Kupffer cells, NKT cells	Increase	Not directly related to ferroptosis; IL-18 is a downstream product of pyroptosis, and its elevation during ferroptosis reflects crosstalk between pyroptosis and ferroptosis rather than a direct regulatory role.	(77, 78)
IL-10	CD4^+^CD25^+^ Tregs, Kupffer cells	Increase (protective negative feedback regulation)	Anti-ferroptotic: In intracerebral hemorrhagic stroke, IL-10 activates IL-10R/STAT3 and upregulates DLK1/AMPK/ACC, reducing lipid ROS and ferroptosis.	(79, 80)

#### 4.1.3 Clinical evidence and biomarkers

Current clinical and translational investigations have delineated the evidentiary profile of ferroptosis in AIH from multiple dimensions, providing clinical-level support for the association between the two. In clinical research, large-scale cohort studies have demonstrated significantly elevated serum ferritin levels in treatment-naïve AIH patients, which exhibit an independent positive correlation with advanced hepatic fibrosis (4, 81). Given that serum ferritin serves dual roles as both an indicator of iron reserves and a non-specific acute-phase reactant, its aberrant elevation not only reflects systemic iron overload but also provides the requisite ferrous ion substrate and pro-oxidant microenvironment essential for ferroptosis initiation (82–84).

Histopathologically, case reports utilizing Perl’s Prussian blue staining have demonstrated marked hepatic iron accumulation in AIH liver biopsy specimens, with severity surpassing that of healthy controls and distribution encompassing hepatocytes, Kupffer cells, and biliary epithelial cells (85, 86). Such tissue-level iron overload can catalyze substantial hydroxyl radical generation via Fenton chemistry, thereby directly driving lipid peroxidation of cellular membranes and establishing the biochemical basis for ferroptosis.

Several caveats must be considered when interpreting these clinical findings. First, ferritin is an acute-phase reactant influenced by inflammatory conditions and metabolic comorbidities including insulin resistance (87, 88); inclusion of HbA1c or fasting glucose as confounding factors would help distinguish AIH-related inflammation from metabolic contributions (89). Second, MDA and 4-HNE are general markers of oxidative stress and end-products of generalized lipid peroxidation—they are not unique to ferroptosis (90). Collectively, these markers should be interpreted as hypothesis-generating circumstantial evidence consistent with a ferroptosis-permissive microenvironment, rather than confirmatory evidence.

#### 4.1.4 Integrated regulatory network and therapeutic prospects

Synthesizing available evidence reveals that patients with AIH exhibit characteristics of intensified oxidative stress, which may coincide with localized disturbances in iron metabolism within specific subgroups or pathological stages, thereby potentially establishing a microenvironment favorable for ferroptosis. The concomitant downregulation of GPX4 and accumulation of lipid peroxidation products mark the initiation of the ferroptotic execution phase.

Viewed through its regulatory architecture, ferroptosis constitutes a hierarchically organized cascade driven by upstream stimuli—exemplified by immune activation in Con A-induced models and putative inflammatory mediators such as S100 proteins—that propagate through multilayered control mechanisms. These encompass pro-ferroptotic pathways, specifically miR-122-5p-mediated suppression of SLC7A11 and stimulator of interferon genes (STING) signaling-driven iron metabolism disruption (6, 91), as well as negative regulatory axes including the A20-Nrf2 pathway and the FGF4-CISD3 pathway (7, 8). Of note, mitochondrial energy metabolism is regulated by thyroid hormones, which modulate GPX4 and lipid metabolism (92); their dysfunction is common in liver disease (93, 94). Thyroid hormone-induced mitochondrial respiration augments ROS production (95), which may intersect with the FGF4-CISD3 axis. Although unexplored in AIH, this endocrine-metabolic link offers a plausible connection between thyroid status and ferroptosis susceptibility. These pathways ultimately converge upon execution phase hallmarks: lipid peroxidation, mitochondrial damage, and hepatocyte death.

This multilayered regulatory network affords multiple potential targets for therapeutic intervention in AIH, encompassing strategies utilizing ferroptosis inhibitors (e.g., Fer-1) and Nrf2 activators for metabolic reprogramming. However, these intervention concepts are currently based on animal model data; their safety, efficacy, and optimal timing in AIH patients remain to be systematically evaluated. Clinically, long-term corticosteroid use in AIH may increase the risk of new-onset diabetes (96), a condition characterized by insulin resistance and compensatory hyperinsulinemia. This hyperinsulinemic state upregulates hepatic SREBP-1c (97), which promotes PUFA synthesis. As PUFAs are substrates for ACSL4/LPCAT3-mediated peroxidation (98), this metabolic state might theoretically sensitize hepatocytes to ferroptosis—a hypothesis that requires experimental verification. Clinicians should be aware of this potential risk. Future clinical investigations are warranted to explore the diagnostic and prognostic value of circulating GPX4, SLC7A11, and other ferroptosis regulatory factors in AIH stratification, and to assess the therapeutic potential of targeting the ferroptosis pathway.

### 4.2 Ferroptosis and cholestatic autoimmune liver diseases: disease-specific mechanisms and translational value of PBC and PSC

**Figure 2:**
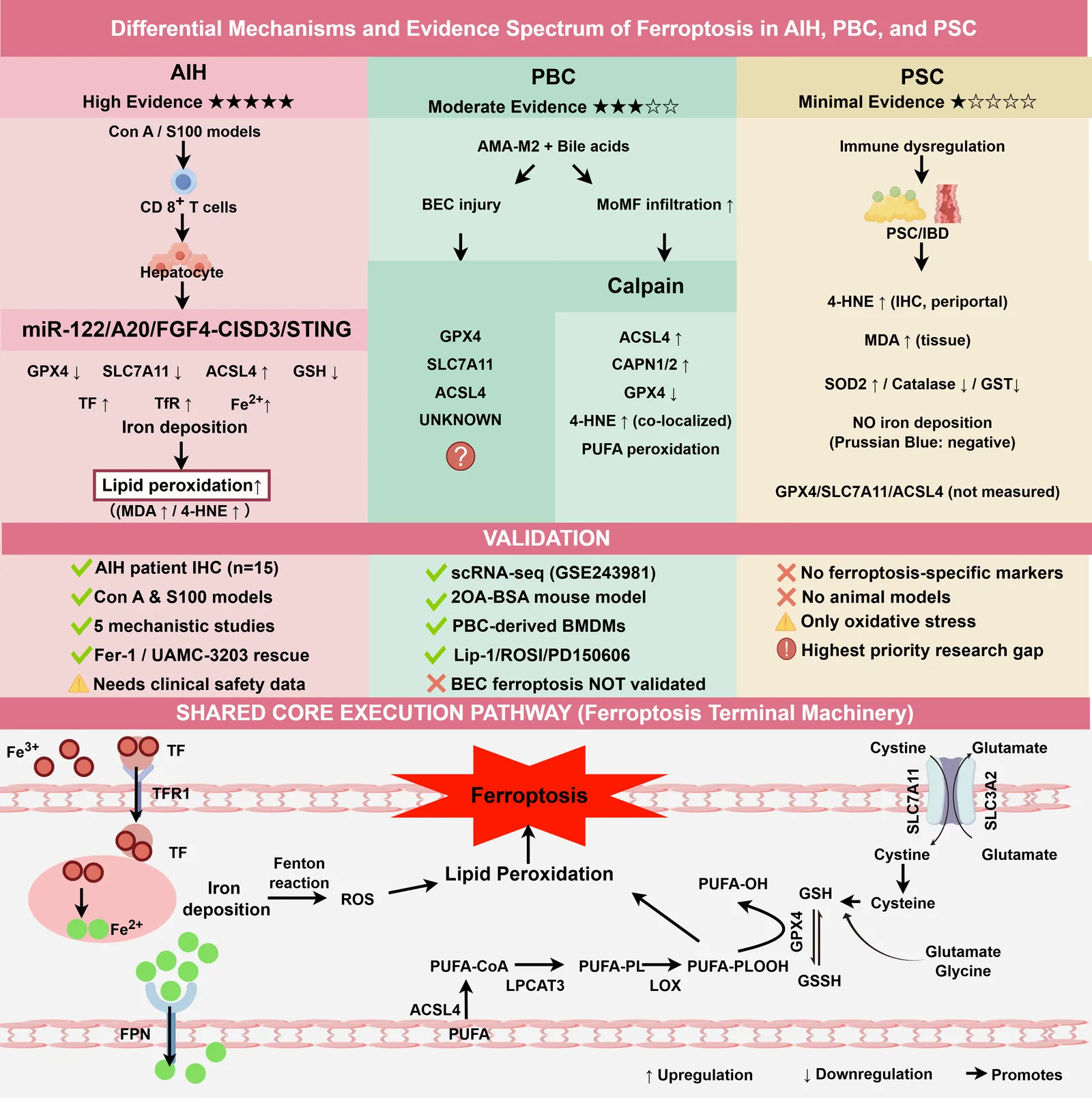
Differential mechanisms and evidence spectrum of ferroptosis in AIH, PBC, and PSC. This figure compares ferroptosis involvement across three autoimmune liver diseases through four vertical layers (triggers, target cell/phenotype, key regulators, and validation evidence) that converge onto a shared terminal execution pathway. This figure was created with Figdraw.

PBC and PSC are both autoimmune liver diseases characterized by chronic cholestasis, but they exhibit striking differences in pathological mechanisms and target cell types. PBC primarily affects intrahepatic small bile ducts, with the core pathology being progressive destruction of biliary epithelial cells (BECs) (99, 100); the disease-specific autoantibody, anti-mitochondrial antibody M2 subtype (AMA-M2), serves as the serological hallmark of PBC, and both AMA-M2-associated immune responses and accumulated bile acids are implicated in the pathogenesis of bile duct injury (101). In contrast, PSC involves a wider range of lesions affecting intrahepatic and/or extrahepatic bile ducts, characterized by fibrous thickening of the bile duct wall and periductal inflammation. Early-stage PSC presents as concentric fibrosis (onion-skin fibrosis), which progressively leads to biliary stricture or obliteration with disease progression, representing a continuous fibrotic process rather than mere stricture formation, and immune-inflammation-driven biliary injury and fibrogenesis are more prominent (102, 103). Despite distinct initiation events, cholestasis, as a shared pathological consequence, may exacerbate hepatic parenchymal damage via diverse cell death pathways (including potential ferroptotic mechanisms), forming a vicious cycle of “immune injury-cholestasis-cell death”. Notably, the underlying molecular mechanisms differ significantly between these two disorders (104, 105). Collectively, AIH, PBC, and PSC exhibit a significant gradient in the strength of evidence for ferroptosis (**Figure 2**), with AIH showing the most abundant evidence, PBC intermediate, and PSC the most limited. The following two sections will discuss the ferroptosis-related evidence in PBC and PSC, respectively.

#### 4.2.1 PBC: cholestatic injury, macrophage ferroptosis, and clinical correlates

The molecular mechanism underlying cholestasis-induced ferroptosis has been preliminarily elucidated in experimental models. In the bile duct ligation (BDL) model, accumulation of hydrophobic bile acids triggers free iron overload in hepatocytes, accompanied by downregulated activity of the Nrf2/HO-1 antioxidant pathway and depletion of the GSH/GPX4 system (106). The ANIT-induced chronic cholestasis model further confirms that liver tissues display typical ferroptotic hallmarks: elevated levels of MDA, reduced mitochondrial cristae and increased mitochondrial membrane density, suggesting that ferroptosis is involved in cholestatic liver injury (107, 108). However, these findings reflect ferroptosis in hepatocytes following obstructive or chemical injury—a context distinct from the immune-mediated bile duct damage in PBC—and should be interpreted as proof-of-principle evidence rather than PBC-specific findings.

Recent studies have provided important clues regarding immune cell ferroptosis in PBC. Liu et al (9) integrated single-cell transcriptomics, multiplex immunofluorescence, and a 2OA-BSA-induced PBC-like mouse model, revealing that MoMFs are significantly expanded in PBC livers and exhibit the most pronounced elevation of ferroptosis module scores among all cell types, with ACSL4 upregulation as a central feature. MoMFs showed markedly enhanced inflammatory crosstalk with biliary epithelial cells, suggesting that MoMF ferroptosis may amplify peribiliary inflammation via paracrine signaling. Pharmacological interventions—including the ferroptosis inhibitor Liproxstatin-1, the indirect ACSL4 inhibitor rosiglitazone, and the calpain inhibitor PD150606—significantly alleviated liver injury, inflammation, and macrophage infiltration in PBC-like mice. Collectively, these findings provide evidence linking ferroptosis to the immune cell compartment in PBC and suggest a macrophage ferroptosis–inflammation amplification loop in disease progression.

Clinically, PBC patients exhibit increased serum MDA and 8-iso-PGF2α levels, a finding that correlates with disease staging, along with detectable 4-HNE adducts in liver tissues and hepatic iron deposition associated with oxidative stress and disease progression (11, 109). Although these observations are consistent with oxidative stress, they are all indirect markers and mainly derived from cholestasis-related models or peripheral blood detection, subject to the same interpretive limitations discussed above for AIH. While preliminary evidence supports MoMF ferroptosis in PBC, direct validation of ferroptosis-specific markers in clinical PBC samples, whether biliary epithelial cells themselves undergo ferroptosis, and its dynamic changes across disease stages remain to be elucidated.

#### 4.2.2 PSC: immune-inflammatory vicious cycle and biliary fibrosis

The core pathogenesis of PSC involves a self-sustaining cycle of aberrant immune activation and biliary epithelial injury. Distinct from the classical antigen-specific recognition pattern, current studies demonstrate that CD4^+^ T cells in PSC patients mainly infiltrate the portal tract, and their activation is likely mediated by recognizing exogenous antigens or autoantibody-biliary epithelial cell complexes, rather than directly recognizing biliary antigens; these immune cells recruit macrophages, dendritic cells and natural killer cells to the portal tract, and secrete pro-inflammatory cytokines such as TNF-α and IL-6 upon activation via pattern recognition receptors, thereby driving the peribiliary inflammatory cascade (110). BECs play a dual role as both target cells and effector cells in this process: injury-induced cytokine release further recruits inflammatory cells, while mediators secreted by infiltrating immune cells continuously aggravate biliary epithelial damage. This forms a vicious cycle of “biliary epithelial destruction–inflammatory infiltration–pro-fibrotic factor release–biliary fibrotic obliteration”, ultimately leading to cholestatic liver injury (111, 112).

Direct evidence linking ferroptosis to PSC remains limited. Shearn et al (113) detected significantly elevated lipid peroxidation products—including 4-HNE, MDA, and acrolein—in liver tissues from PSC patients with concurrent IBD, with predominant localization to hepatocytes and biliary epithelial cells in portal tracts and around fibrotic areas. Concurrently, the antioxidant system exhibited dysregulation characterized by downregulated GPx1, catalase inhibition, and an approximately 30% reduction in total GST activity, with upregulation of SOD2 as a potential compensatory response to oxidative stress. However, this study did not examine GPX4, SLC7A11, or ACSL4 expression, and Prussian blue staining revealed no evident iron deposition in PSC livers, suggesting that the lipid peroxidation may primarily derive from inflammation-mediated oxidative injury rather than iron-dependent Fenton chemistry.

Thus, although PSC displays oxidative stress alterations that overlap with the ferroptotic phenotype, direct evidence for the execution phase of ferroptosis remains lacking. Theoretically, a complex crosstalk exists between ferroptosis and inflammation: ferroptosis can trigger inflammatory responses by releasing DAMPs, and exacerbate inflammation via oxidative stress and lipid peroxide accumulation; conversely, inflammation can promote ferroptosis by facilitating iron overload and lipid peroxidation (114–116). This bidirectional positive feedback mechanism provides a theoretical basis for the pathological role of ferroptosis in various inflammatory diseases. However, a critical distinction must be made: although the ferroptosis-inflammation crosstalk has been verified in multiple disease models, direct evidence from clinical samples or animal models supporting its specific role in PSC-related biliary epithelial injury is currently lacking. Unlike AIH—in which multiple regulatory nodes have been identified in animal models—and PBC—in which preliminary confirmatory evidence has been obtained at the MoMF level—the association between PSC and ferroptosis has not yet been directly demonstrated experimentally. Current PSC research focuses more on the core roles of BECs senescence and activated secretory phenotype in disease progression (103, 116). Therefore, the specific role of ferroptosis in PSC remains to be further elucidated in future studies, and the exploration of related intervention strategies requires sufficient experimental validation.

#### 4.2.3 Translational value: theoretical potential and research limitations

Based on the ferroptotic mechanisms of PBC and PSC, their translational application value is currently at a critical stage transitioning from basic research to clinical validation. Current human evidence is mainly derived from cross-sectional cohort studies and immunohistochemical detection of hepatic oxidative stress markers. Although these findings preliminarily indicate the involvement of ferroptosis in disease progression, prospective validation cohorts and interventional clinical trials are still lacking. This evidence status not only reflects the emerging nature of ferroptosis research in cholestatic liver diseases, but also highlights the urgency and research opportunities of establishing disease-specific biomarker systems.

For therapeutic strategies, the standard treatments for PBC are ursodeoxycholic acid (UDCA) and obeticholic acid (OCA), while specific pharmacotherapies for PSC are lacking. Targeted interventions against ferroptosis are still theoretical speculations based on mechanistic insights, with no preclinical or clinical validation data available globally. Nevertheless, this research gap precisely provides a direction for exploring novel therapeutic strategies: compared with the established UDCA therapy, the potential theoretical advantage of ferroptosis-targeted interventions lies in their ability to block early biliary epithelial damage, offering new targets for disease-modifying therapy. Future large-scale prospective clinical studies are required to validate the diagnostic efficacy of potential biomarkers, discover more specific novel biomarkers via multi-omics techniques, and verify the safety and effectiveness of ferroptosis-targeted interventions through rigorous preclinical models and early-phase clinical trials, so as to achieve the leap from mechanistic research to clinical translation.

## 5 Targeting ferroptosis: precise intervention strategies based on AIH-specific regulatory networks

**Figure 3:**
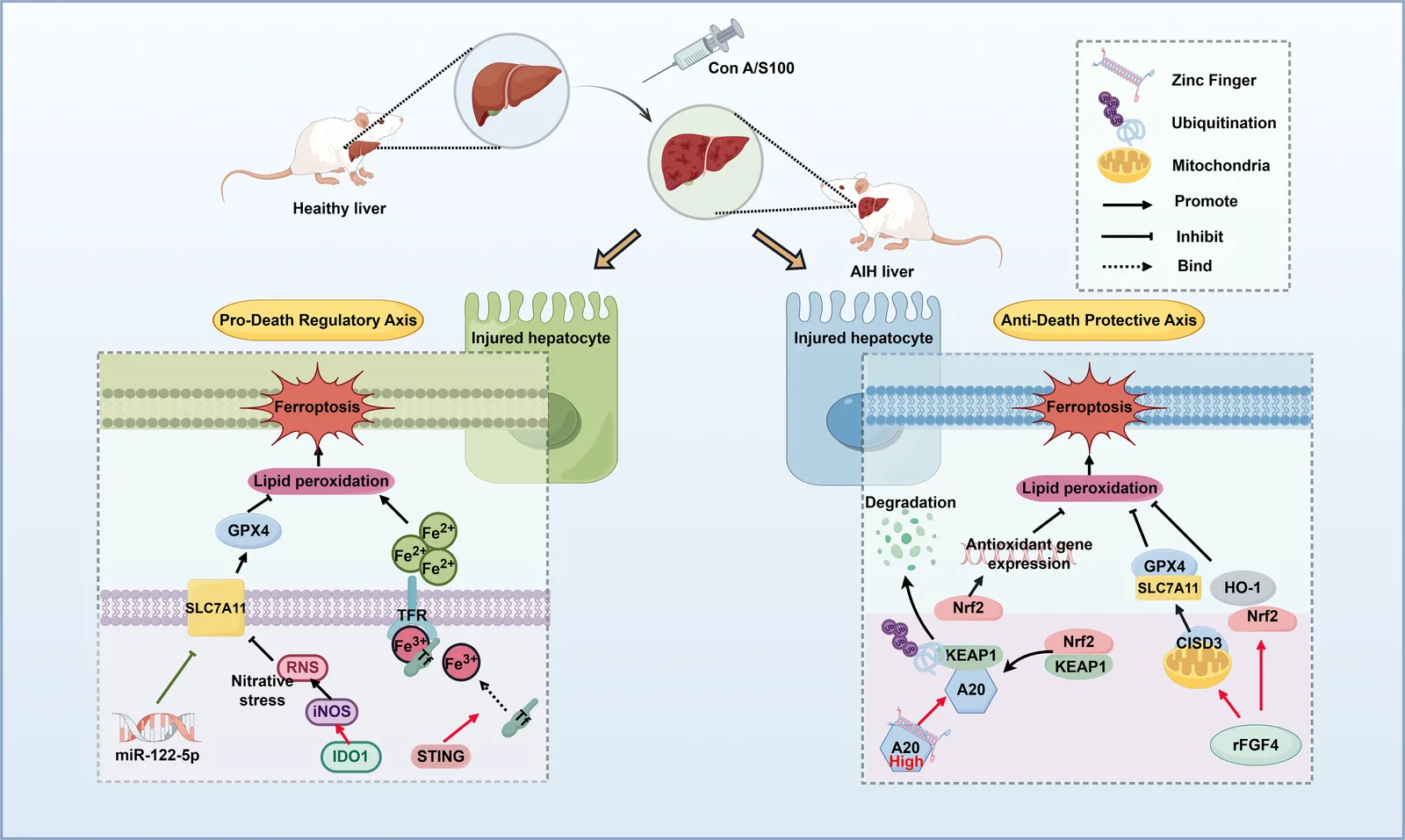
Bidirectional regulatory network of ferroptosis in AIH hepatocytes. This figure summarizes the balanced regulation of pro-death and anti-death signals in AIH hepatocytes. Left panel (pro-death axis): miR-122-5p targets and inhibits SLC7A11, impairing cystine uptake and GSH synthesis; IDO1/iNOS suppresses SLC7A11 through nitrosative stress; STING activation promotes iron accumulation. Right panel (anti-death axis): A20 mediates K48-ubiquitination and degradation of KEAP1, releasing NRF2 to activate transcription of antioxidant genes; rFGF4 upregulates CISD3 to suppress mitochondrial ROS generation, while also activating the Nrf2/HO-1 pathway to enhance antioxidant defense. This figure was created with Figdraw.

Existing studies have confirmed that the broad-spectrum ferroptosis inhibitor Ferrostatin-1 can alleviate liver injury in AIH animal models through mechanisms such as upregulating GPX4 and downregulating ACSL4 (47, 117). However, this intervention only targets the common terminal execution stage of the ferroptosis pathway and has not fully reflected the unique pathological characteristics that distinguish AIH from other types of liver diseases. Based on the latest evidence summarized in this review, ferroptosis in AIH is controlled by a set of specific bidirectional regulatory networks (**Figure 3**), and these molecular nodes are expected to become more disease-specific intervention targets.

### 5.1 Screening of ferroptosis-specific molecular regulators uniquely associated with AIH

The core execution mechanisms of ferroptosis are highly conserved in various organ injuries, but AIH is characterized by disorders of disease-specific upstream regulatory nodes. These nodes regulate the activation threshold and progression rate of the conserved pathway, which not only constitute the molecular characteristics that distinguish AIH from other liver diseases, but also provide specific targets for precise intervention.

In terms of pro-death regulatory nodes, miR-122-5p, as a liver-specifically enriched microRNA, is significantly upregulated in the serum and liver tissues of AIH patients (118). This molecule directly targets and inhibits the translation of SLC7A11, impairs the ability of hepatocytes to take up cystine and synthesize glutathione, and thereby reduces the antioxidant defense mediated by GPX4 (6). Notably, the liver-high expression characteristic of miR-122-5p provides a natural advantage for organ-targeted intervention, and this regulatory abnormality is a unique post-transcriptional pathological feature of AIH, rather than a universal attribute of the ferroptosis pathway (119). Meanwhile, STING signaling disrupts iron homeostasis in AIH models, leading to an increase in intracellular free iron and thereby inducing ferroptosis. This mechanism directly links innate immune activation to iron metabolism disorders, forming a specific link of immune-metabolic crosstalk in AIH (91).

Corresponding to the abnormal enhancement of the aforementioned pro-death signals, the anti-death protective mechanism of AIH exhibits a feature of functional inactivation. A20, as a ubiquitin-editing enzyme induced by inflammatory signals, indirectly promotes Nrf2 protein stability and its nuclear translocation by mediating K48-ubiquitination and degradation of KEAP1, thereby activating the transcription of downstream antioxidant genes. However, the activity of this pathway is significantly downregulated in AIH lesions, leading to impaired KEAP1 degradation, inhibition of Nrf2 signaling, and failure of the endogenous antioxidant barrier, resulting in a dual imbalance characterized by enhanced pro-death signals and weakened anti-death function (7). Furthermore, FGF4 exhibits a protective upregulation in AIH models, and it suppresses ferroptosis and alleviates hepatic inflammation by upregulating the expression of the mitochondrial membrane protein CISD3. Overexpression of CISD3 enhances the resistance of hepatocytes to ferroptosis, whereas CISD3 knockdown reduces the levels of ferroptosis markers including SLC7A11 and GPX4, indicating that CISD3 plays a pivotal role in regulating the ferroptosis sensitivity of hepatocytes. In AIH, impairment of this protective signaling axis gives rise to CISD3 downregulation and elevated ROS production, which in turn accelerates mitochondria-dependent ferroptosis, suggesting that restoring this pathway may block the early stage of the cascade reaction. In addition to its role in mitochondrial regulation, recombinant fibroblast growth factor 4 (rFGF4) also enhances the antioxidant defense capacity of hepatocytes by activating the Nrf2/HO-1 signaling pathway (8). The aberrant activation of these pro-death nodes and the functional inactivation of anti-death mechanisms collectively contribute to the bidirectional imbalance characteristic of ferroptosis regulation in AIH, which also provides definite molecular targets for targeted intervention.

### 5.2 Precise intervention strategies based on AIH-specific regulatory networks

The imbalance of the aforementioned bidirectional regulatory network provides multiple actionable entry points for the precise intervention of AIH. In response to upstream regulatory aberrations, antisense oligonucleotides or locked nucleic acid-modified inhibitors can be designed to block miR-122-5p and restore the physiological function of System Xc⁻. The core superiority of this strategy resides in the natural organ-targeting property endowed by the liver-specific high expression of miR-122, which helps minimize systemic side effects and fulfills the therapeutic goal of precise intrahepatic regulation with negligible extrahepatic interference (6, 120). As for iron metabolism disorders, the combined administration of iron chelators and STING pathway inhibitors merits further exploration. By blocking ferritinophagy and subsequent free iron release, this strategy fundamentally attenuates Fenton reaction-driven lipid peroxidation at the source, and integrating immune modulation with iron metabolism intervention enables etiological-level precise blockade (121, 122). Additionally, targeting the inactivation of endogenous protective machineries, Nrf2 agonists or A20 expression enhancers can be adopted to rebuild the antioxidant defense capacity of hepatocytes. Unlike strategies that merely exert exogenous blockade on death signals, this approach focuses on restoring endogenous protective mechanisms, which aligns with the therapeutic rationale of strengthening vital qi to eliminate pathogenic factors (123, 124). For the early triggering cascade of ferroptosis, liver-targeted delivery systems of recombinant FGF4 protein or FGF4 receptor agonists can be developed to suppress ROS generation by upregulating mitochondrial CISD3 expression, thereby mitigating mitochondrial-derived oxidative stress damage. Given the pivotal role of CISD3 in governing hepatocyte ferroptosis sensitivity, such intervention is expected to prevent the amplification of the ferroptotic cascade, yet the optimal intervention window (e.g., preemptive administration during the metabolic stress phase) still remains to be validated by further experimental studies (125, 126). These strategies form an intervention system covering different stages of the ferroptotic cascade from four dimensions: post-transcriptional regulation, iron metabolism intervention, antioxidant restoration, and mitochondrial protection.

### 5.3 Stage-specific precision intervention framework for AIH

Based on the above framework, the disease course of AIH can be divided into three functional phases: acute active, remission maintenance, and cirrhosis. In the acute phase, STING-driven iron accumulation (91), miR-122-5p-mediated SLC7A11 suppression (6), and impaired A20–NRF2 axis (7) collectively lower the hepatocyte ferroptotic threshold, creating a “high-risk window” for ferroptosis execution. In the remission phase, although immune attack is controlled, endogenous antioxidants (A20, NRF2, CISD3) remain deficient, rendering hepatocytes vulnerable to residual oxidative stress and predisposing to relapse and chronic injury (7, 8). In the cirrhosis phase, chronic iron overload, antioxidant depletion, and declining regenerative capacity allow ferroptosis to accelerate hepatic decompensation via hepatic stellate cell activation and fibrosis (113).

**Figure 4:**
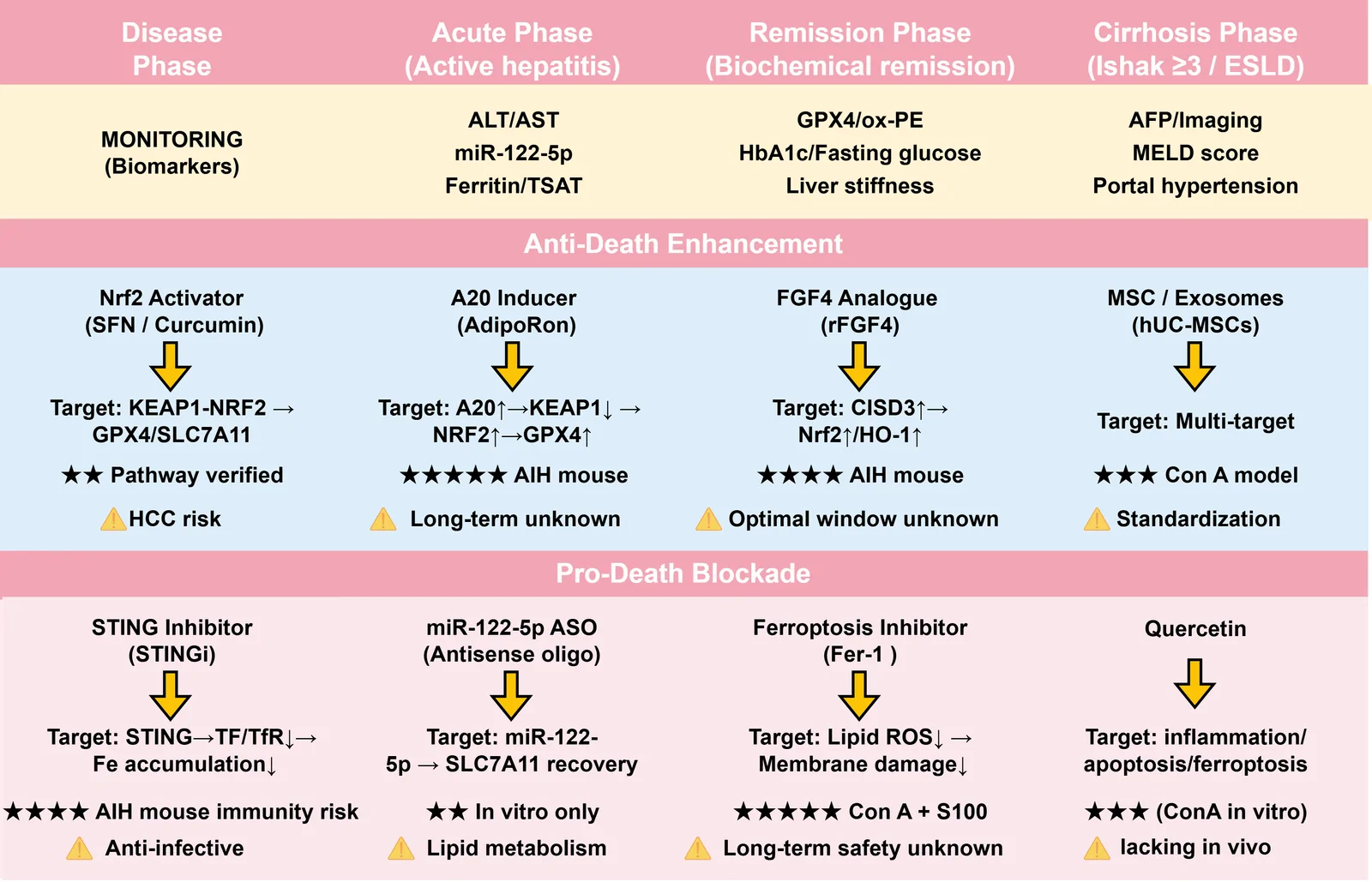
Framework for stage-specific monitoring and ferroptosis-targeted interventions in AIH. The figure stratifies AIH progression into three functional phases (acute, remission, cirrhosis) and assigns phase-matched biomarker panels for disease activity, iron burden, metabolic safety, and portal hypertension. A range of therapeutic modalities targeting ferroptosis is presented, including STING inhibitor, A20 inducer, FGF4 analogue, Nrf2 activator, ferroptosis inhibitor (Fer-1), MSC/exosomes, and quercetin. Each intervention is annotated with evidence level and key translational limitations. This figure was created with Figdraw.

Based on these stage-specific characteristics, **Figure 4** allocates intervention strategies by phase across two integrated layers: biomarker monitoring and therapeutic intervention. In the monitoring layer, acute-phase biomarkers assess disease activity and iron burden; remission-phase markers monitor residual ferroptosis and metabolic safety; cirrhosis-phase indicators include AFP/imaging surveillance, MELD score, and portal hypertension assessment. Anti-death enhancement strategies are primarily suited for remission maintenance, while mesenchymal stem cells (MSCs)/exosomes (127) require standardization. Pro-death blockade strategies are phase-dependent: STING inhibitors are prioritized in the acute phase for upstream iron blockade; broad-spectrum ferroptosis inhibitors (Fer-1) are better reserved for remission maintenance due to their terminal site of action and unknown long-term safety; quercetin (128) and miR-122-5p antisense oligonucleotides require further *in vivo* validation.

Thus, ferroptosis-targeted interventions should be dynamically adjusted by disease phase—acute phase prioritizing upstream blockade, remission phase focusing on systemic restoration, and cirrhosis phase requiring careful benefit-risk assessment. All strategies face common translational obstacles: lack of humanized PK/PD data and unknown drug-drug interactions with standard AIH therapy (glucocorticoids/azathioprine).

### 5.4 Future research directions and key issues to be addressed urgently

Building upon these translational obstacles, safety concerns warrant particular attention before clinical translation. Chronic miR-122-5p inhibition may interfere with normal hepatic lipid and fatty acid metabolism; systemic STING blockade could increase viral susceptibility or compromise tumor immune surveillance; Nrf2 hyperactivation has been associated with tumorigenesis and may affect pharmacokinetics of concomitant medications via upregulation of drug-metabolizing enzymes; and the FGF4/CISD3 axis, involved in mitochondrial regulation, carries unknown long-term risks for extrahepatic high-energy organs. Importantly, these concerns currently rest on mechanistic inference and short-term animal data, with no human evidence regarding long-term safety, immunogenicity, or drug-drug interactions. Thus, systematic toxicological and pharmacokinetic evaluations in non-human primate models, followed by rigorously designed early-phase trials, are essential to validate safety and tolerability.

### 5.5 Broader translational challenges and open questions

Beyond safety and preliminary validation, several broader translational issues demand systematic resolution. At the disease characterization level, the temporal expression dynamics of these molecules and their correlation with immune phenotypes need validation through serial sampling from AIH patient cohorts—a prerequisite for designing stage-specific interventions. At the therapeutic strategy level, the stage-adapted framework requires prospective validation to define biochemical thresholds for phase transition, assess safety boundaries of combined strategies, and determine whether molecular subtype-guided regimens outperform conventional stepwise approaches. At the translational application level, non-invasive biomarkers—including circulating miR-122-5p levels or specific oxidized phospholipid profiles—must be developed to enable molecular typing-based precision therapy. Only through integrated progress across these dimensions can these strategies translate from mechanistic insights into tangible clinical benefits.

## 6 Conclusion and outlook

The core conclusion of this review is that the pathological mechanism of ferroptosis in AIH has been elucidated by multi-layered evidence, forming a coherent evidence framework from clinical phenotypes (elevated serum ferritin, hepatic iron deposition) to molecular mechanisms (inhibition of the miR-122-5p/SLC7A11 axis, STING-mediated iron metabolic disorder, inactivation of the A20-Nrf2 and FGF4-CISD3 protective axes). These liver-specific regulatory nodes, through the bidirectional imbalance between enhanced pro-death signaling and inactivated anti-death function, constitute the core mechanism that distinguishes AIH from other liver diseases. Among them, the liver-enriched property of miR-122-5p provides a natural targeting advantage for precise intervention. In contrast, current research on PBC mainly remains at the level of association between oxidative stress markers and ferroptosis phenotypes, with emerging evidence of ACSL4 upregulation in MoMFs; however, direct evidence for ferroptosis execution in biliary epithelial cells remains absent, and mechanistic validation in this context is still lacking. The association between PSC and ferroptosis is even weaker, with most existing studies being theoretical speculations, lacking evidence of altered expression of ferroptosis regulatory factors in patient samples, and mechanistic verification specifically linking these observations to ferroptosis execution remains lacking.

Future research should be carried out along two main lines: at the basic level, there is an urgent need to establish a diagnostic standard system to distinguish “confirmation” from “speculation”. Conduct multi-center prospective cohort studies in AIH to verify the prognostic value of markers such as serum GPX4 and oxidized phospholipid profiles; extend the current MoMF-focused findings to biliary epithelial cells and validate the translational potential of targeting the calpain/ACSL4 axis in PBC; construct biliary organoid models for PSC to obtain direct mechanistic evidence. At the translational level, implement staged precise interventions for AIH: prioritize blocking upstream pro-death signals STING-mediated iron dysregulation and the miR-122-5p/SLC7A11 axis in the acute phase, and focus on activating endogenous defense functions such as the A20-Nrf2 axis in the remission phase; develop non-invasive markers such as circulating miR-122-5p and oxidized phospholipid profiles to guide molecular typing; verify the efficacy and safety of targeting strategies in humanized models. Only by strictly distinguishing established findings from hypotheses to be verified and constructing an evidence-based translational path can ferroptosis research truly bring breakthroughs to the prevention and treatment of autoimmune liver diseases.
